# ssPINE: Probabilistic Algorithm for Automated Chemical Shift Assignment of Solid-State NMR Data from Complex Protein Systems

**DOI:** 10.3390/membranes12090834

**Published:** 2022-08-26

**Authors:** Adilakshmi Dwarasala, Mehdi Rahimi, John L. Markley, Woonghee Lee

**Affiliations:** 1 Department of Chemistry, University of Colorado Denver, Denver, CO 80217, USA; 2 Department of Biochemistry, University of Wisconsin-Madison, Madison, WI 53706, USA

**Keywords:** solid-state NMR, membrane proteins, assignment, automation, ssPINE, MAS-NMR

## Abstract

The heightened dipolar interactions in solids render solid-state NMR (ssNMR) spectra more difficult to interpret than solution NMR spectra. On the other hand, ssNMR does not suffer from severe molecular weight limitations like solution NMR. In recent years, ssNMR has undergone rapid technological developments that have enabled structure–function studies of increasingly larger biomolecules, including membrane proteins. Current methodology includes stable isotope labeling schemes, non-uniform sampling with spectral reconstruction, faster magic angle spinning, and innovative pulse sequences that capture different types of interactions among spins. However, computational tools for the analysis of complex ssNMR data from membrane proteins and other challenging protein systems have lagged behind those for solution NMR. Before a structure can be determined, thousands of signals from individual types of multidimensional ssNMR spectra of samples, which may have differing isotopic composition, must be recognized, correlated, categorized, and eventually assigned to atoms in the chemical structure. To address these tedious steps, we have developed an automated algorithm for ssNMR spectra called “ssPINE”. The ssPINE software accepts the sequence of the protein plus peak lists from a variety of ssNMR experiments as inputs and offers automated backbone and side-chain assignments. The alpha version of ssPINE, which we describe here, is freely available through a web submission form.

## 1. Introduction

NMR spectroscopy is one of the major biophysical methods, along with X-ray crystallography [1,2] and cryo-electron microscopy [3], for determining structures of biomolecules. NMR is used to study structure–function relationships of membrane proteins and large macromolecular assemblies [4] along with their interactions with small molecules [5] as an approach to drug discovery [6].

Both solution and solid-state NMR techniques provide important information about the structures and dynamics of membrane proteins [7,8]. Solid-state NMR (ssNMR) with magic angle spinning (MAS) has advantages over solution NMR for studies of large and immobilized proteins [9,10]. Anisotropic nuclear spin interaction information from ssNMR can be extremely useful for structure determination and dynamics [11,12]. The orientation of regions of membrane proteins can be extracted from ssNMR spectra of mechanically- or magnetically-aligned membranes [13]. The broad lines and low resolution of ssNMR spectra resulting from anisotropy can be overcome in part by ultra-high MAS, cross-polarization, refined pulse sequences [14], and non-uniform sampling (NUS). Ultra-high-field NMR spectrometers operating at 1.1 GHz and 1.2 GHz are improving the resolution and sensitivity of ssNMR spectra of membrane proteins and their complexes. The above-mentioned methods are enabling the collection of improved spectral data, but manual analysis of the data to obtain chemical shift assignments and structural constraints is tedious because thousands of signals need to be analyzed, correlated, and labeled.

Software technology has reduced the burden of analyzing data from solution NMR studies of biomolecules. Available web-based resources provide automated and semi-automated algorithms for determining different parameters of biomolecules and their structure [15,16,17,18]. We recently developed an updated version of the assignment engine PINE [19], I-PINE (Integrative Probabilistic Interaction Network of Evidence) [20], which utilizes a Bayesian-based probabilistic interaction network. I-PINE supports a larger range of NMR experiments and integrates real-time statistical analysis of the PACSY database [21]. The I-PINE web server produces higher assignment coverage and accuracy than PINE and supports structure determinations based on chemical shift assignments. The POKY suite includes iPick [22], for peak picking and cross-validation of peaks from different spectra, I-PINE, and PINE-SPARKY.2 [23], a user-friendly graphical user interface (GUI) for submitting, importing, and validating the data [24].

For ssNMR data, PISA-SPARKY [25], a plugin for the assignment program, NMRFAM-SPARKY [26], supports the analysis of data from oriented samples [27]. PISA-SPARKY, along with its features, are now included in the POKY suite. Recently, the Veglia group introduced “a one-shot approach” called PHORONESIS, which generates up to ten 3D ^1^H-detected ssNMR spectra [28]. They used the I-PINE webserver to analyze the spectra, and found that the yield of sequential assignments was similar to that for solution NMR data. The Hunter Moseley and Chad Rienstra groups developed an ssNMR version of AutoAssign and demonstrated its ability to assign ssNMR data from the small protein, GB1 [29]. The software returned 84.1% correct assignments. The ssFLYA algorithm, which was introduced by Schmidt and colleagues [30], and is currently available for only commercial users, yielded 88–87% and 77–90% correctness on protein microcrystals and amyloids.

Here, we describe ssPINE (solid-state PINE), a software package that is designed to handle the challenging features of ssNMR data from membrane proteins and other complex protein systems. ssPINE accepts, as inputs, 2D and 3D ssNMR data and gives, as an output, chemical shift assignments and their probabilistic correctness. We have evaluated the performance of ssPINE with data from GB1 and with additional protein NMR data from the BMRB database [31]. The alpha version of ssPINE is freely available through a web server utility at https://poky.clas.ucdenver.edu/ssPINE.

## 2. Materials and Methods

### 2.1. ssPINE Algorithm

**Figure 1:**
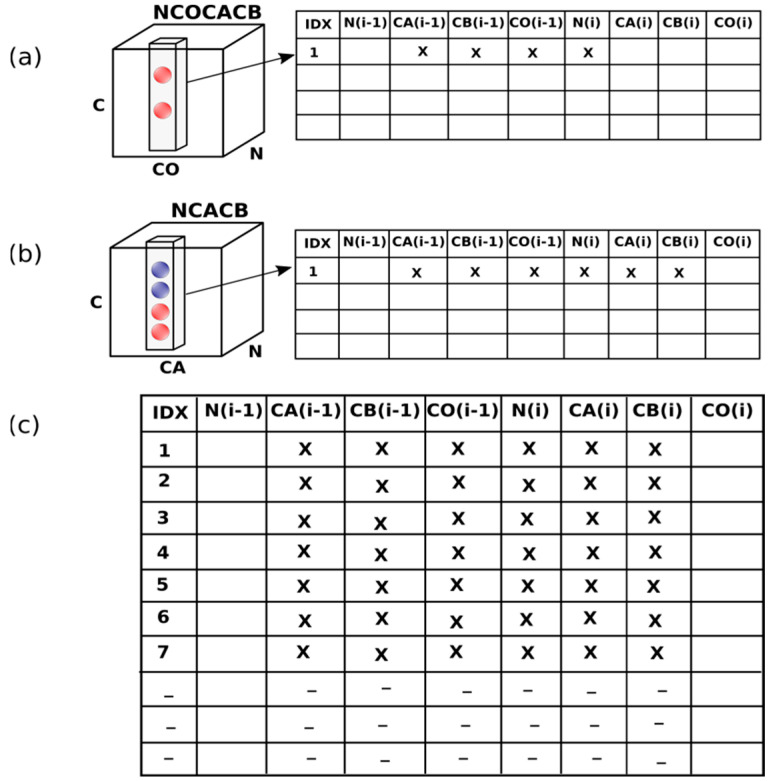
Spin system matrix assembly in ssPINE. (**a**) Peaks from the strip of the NCOCACB experiment containing the CA(i − 1) and CB(i − 1) resonances are inserted in the row of the table corresponding to the ith residue (IDX). Note that the peak is selected from the CO(i − 1) and N(i), in root information. (**b**) Similarly, peaks from the strip of the NCACB experiment containing the CA(i) and CB(i) resonances are inserted in the row of the table corresponding to the ith residue. N(i) and CA(i) in root information are used to select a peak from NCACB. (**c**) The process is repeated for all residues in the peptide sequence.

As its first step, ssPINE generates spin system matrices [32], as shown in Figure 1. The main difference between the I-PINE and ssPINE algorithms is in their approach to comparing peaks from different experiments. I-PINE uses N*_i_* and H*_i_* in root experiments to find correlated signals (CA/CB/CO*_i_*_−1_, CA/CB/CO*_i_*) in different experiments and to establish di-peptide arrays {CA/CB/CO*_i_*_−1_ N*_i_* CA/CB/CO*_i_*}. Then, it establishes a vector [CA*_i_*_−1_, CB*_i_*_−1_, CO*_i_*_−1_, N*_i_*, CA*_i_*, CB*_i_*, CO*_i_*] and compares it to [CA*_i_*_−1_, CB*_i_*_−1_, CO*_i_*_−1_] and [CA*_i_*, CB*_i_*, CO*_i_*] from the other di-peptide arrays. Finally, I-PINE compares [CA*_i_*_−1_, CB*_i_*_−1_, CO*_i_*_−1_] to [CA*_i_*, CB*_i_*, CO*_i_*] in all vectors. By contrast, ssPINE uses CO*_i_*_−1_, N*_i_*_,_ and CA*_i_* in root experiments to find di-peptide signals (CX*_i_*_−1_, CX*_i_*). If an experiment is providing CO*_i_*_−1_, N*_i_*_,_ and CA*_i_*, but a single peak is not provided (e.g., CANCO), ssPINE combines information from different experiments, such as NCOCACB and NCACB, to obtain these correlations. Unlike I-PINE, ssPINE generates each spin system matrix by iterated spectral resolution steps (tolerances) using inter-residue connectivities optimized by a probability approach. This is a basic and important component of the ssNMR algorithm because it helps to overcome the variable spectral resolution of ssNMR spectra. ssPINE calculates the quality of the data at each step until it reaches the point where there is no further improvement in the spin system matrix. If the quality of the data is above a threshold value, as determined by the number of spin systems and correlations between spin systems is identified compared to the numbers expected, then the process continues to the pentapeptide generation step. Otherwise, the process terminates and informs the user that more information is required. The pentapeptide generation step, which assigns signals to atoms in sequences of five amino acid residues, finds the best marginal probabilities by using the belief propagation algorithm [33] to evaluate relations between spin systems. This step includes the identification of secondary structural elements, the evaluation of possible referencing errors, and the continued assignment of backbone spin systems until convergence is reached or, alternatively, until the specified number of iterations has occurred. The last step utilizes the Bayesian network model of PINE and I-PINE to assign side chain signals [16,20]. See the Supporting Information for a detailed description of the ssPINE algorithm.

### 2.2. Input Files

As with PINE and I-PINE, peak lists (either raw or refined) and sequence files are used as inputs to ssPINE. The supported solid-state NMR experiments and their profiles are shown in Table 1. The minimum set of peak lists for assignments are those from 2D-CC, 2D-NCA, 2D-NCO, 3D-NCACX, 3D-NCOCX, and CAN(CO)CX ssNMR experiments. Data from additional ssNMR experiments can be added to improve the accuracy and completeness of the results.

#### 2.2.1. Preparation of Peak Lists

Several peak list formats are accepted: Sparky (UCSF-/NMRFAM-SPARKY or POKY) with the *.list* file extension prepared in the peak list window (two-letter-code “lt” with the *Data Heights* option turned on), XEASY with the *.peaks* file extension [34], nmrDraw with the *.ft2* file extension, NMRView with the *.xpk* file extension, and I-PINE with the *.txt* file extension. The file extension in the file name should match its actual format. Other programs can generate the Sparky format, which is one of the most common file formats in the field. For example, CARA has the *WriteSparkyPeakList.lua* script, and CCPNMR v2 has the *Format Converter* program [35,36]. The POKY suite contains multiple options for generating peak lists; of these, one of the easiest approaches is iPick. With iPick, the user simply selects one or more spectra from the session and clicks on the “Run iPick” button. After peak lists have been generated for each spectrum, the “Peak List” window opens, and by clicking on the “Save” button, the user can designate the names for the peak lists. Peak lists can be refined by hand or by software to remove noise or other spurious peaks.

#### 2.2.2. Protein Sequence

ssPINE accepts peptide sequences in either one- or three-letter amino acid codes as ASCII text files. Sequences submitted in RTF (Rich Text Format; *.rtf*), ODT (OpenDocument Text; *.odt*), or DOCX (Office Open XML; *.docx*) are automatically converted to ASCII.

### 2.3. Output Files

**Figure 2:**
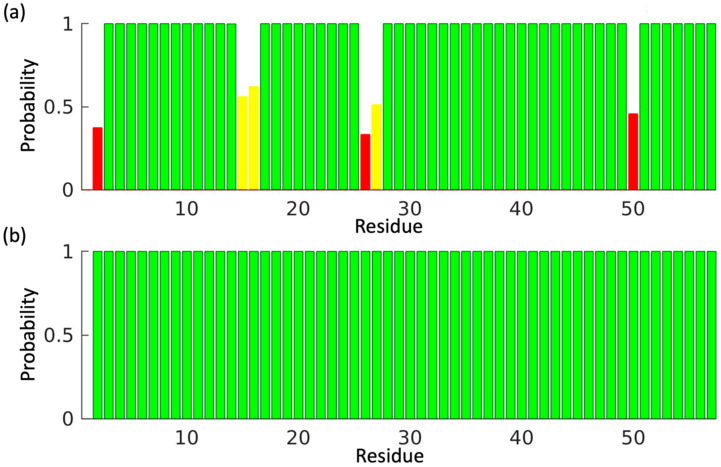
Bar graphs indicating the correct assignment probability (*p*) for each residue of GBI resulting from ssPINE analysis. Green indicates *p* *greater than* 0.99; cyan indicates *p* = 0.85–0.99; yellow indicates *p* = 0.5–0.84; red indicates *p* *less than* 0.5; and gray indicates no assignment (not seen with these test sets). (**a**) Unrefined GBI data as input. (**b**) Refined GBI data as input.

The ssPINE output consists of several files: (1) The list of ssNMR experiments used. (2) A bar graph indicating the assignment probabilities of each residue in the protein (Figure 2). (3) Separate files with the backbone and sidechain chemical shift assignments of each residue of the protein in NMR-STAR 2.1 and 3.1 formats. (4) Sparky format assignment labels and frequencies. (5) Protein secondary structure prediction by PECAN (Protein Energetic Conformational Analysis from NMR chemical shifts) [37]. (6) Chemical shift referencing errors in each experiment, as detected by LACS (Linear Analysis of Chemical Shifts) [38], which is used in redefining offsets during the assignment iteration. (It is recommended that the user use these values to correct the offset for each peak list when a job is resubmitted. This will reduce the computational time and improve the assignment accuracy).

### 2.4. Data Used in Developing ssPINE

#### 2.4.1. Data from GB1

In the early stages of developing ssPINE, we used unpublished ssNMR data from the uniformly ^13^C/^15^N-labeled small (56 residue, 6.2 kDa) protein GB1 that was generously provided by Chad Rienstra’s group. GB1, which is the streptococcal B1 immunoglobulin-binding domain of protein G20, has been used frequently as a standard sample in the development of NMR technology. We prepared both unrefined and refined peak lists from raw data from the following ssNMR experiments: 2D-CC, 2D-NCA, 2D-NCACB, 2D-NCO, 3D-NCACB, 3D-NCACX, 3D-NCACO, 3D-CANCO, 3D-CANCOCX, 3D-NCOCA, 3D-NCOCACB, and 3D-NCOCX. We prepared unrefined peak lists automatically with the iPick peak picking tool of POKY (two-letter-code *iP*). Subsequently, we created refined peak lists by using the cross-validation tool of iPick to weed out noise and non-sequential signals.

#### 2.4.2. Other Protein NMR Data

Additional data from the PACSY database [21] were used in refining and optimizing the ssPINE algorithm. PACSY is a relational database that contains post-processed information from BMRB [39] and PDB [40]. Data from 82 proteins, including both large (181 residues) and small (26 residues) proteins, were included (SI Table S1). Most datasets were from solution NMR, because few ssNMR entries in the BMRB contain complete assignments. We created synthetic peak lists for 2D-CC, 2D-NCA, 2D-NCACB, 2D-NCO, 3D-NCACB, 3D-NCACX, 3D-NCACO, 3D-CANCO, 3D-CANCOCX, 3D-NCOCA, 3D-NCOCACB, and 3D-NCOCX ssNMR spectra of these proteins. For a more controlled evaluation, we only regarded sequential cross-peaks.

### 2.5. ssPINE Web Server

We utilized multiple technologies in implementing the ssPINE algorithm as a web server. Programs written in Perl, Python, and shell scripting handle various parts of the task. A web-facing server hosts a form that the user can fill out with their information: the amino acid sequence file and the peak lists from specified 2D and 3D solid-state NMR experiments. By clicking the “Submit” button, this information is validated and sent to a processing server. After the automated backbone and sidechain assignments are completed, the result is sent back to the user’s email address. From there, the user can download all the result files. The actual running time is determined by the size of the protein and the complexity of the problem, including peak list quality provided by the user, but jobs usually require less than one hour. The ssPINE web server is hosted at the University of Colorado, Denver and is accessible at: https://poky.clas.ucdenver.edu/ssPINE. No login or signup is required, and the server is open to all researchers at no cost and processes submissions in the order in which they are received.

## 3. Results

We evaluated the results with GB1 in terms of their completeness and correctness. “Completeness” is the number of automatically-assigned chemical shifts by ssPINE divided by the number of assignments for GB1 derived from our manual assignment of the ssNMR data. “Correctness” is the number of correct assignments made by ssPINE divided by the number of manual assignments. Given that ssPINE provides multiple assignment candidates with associated probabilities, only the assignment candidate with the highest probability is used in the evaluation of completeness and correctness.

With the unrefined peak lists of GB1 as inputs, ssPINE yielded 100% (219/219) completeness and an average of 97.26% (213/219) correctness for the backbone chemical shift assignments (Figure 2a). With the refined peak lists of GB1, ssPINE yielded 100% (219/219) completeness and 100% (219/219) accuracy (Figure 2b).

We also tested ssPINE algorithm with synthetic peak lists from other proteins whose assigned chemical shifts had been deposited in BMRB (see Section 2.4.2). These BMRB assignments are assumed to be correct and were used in evaluating the correctness of the ssPINE results. The numbers of BMRB and ssPINE assignments were used, respectively, as the denominator and numerator in the completeness calculation. The number of valid ssPINE assignments (“given” assignments) at the different probability cutoffs were used as the denominator in the correctness calculation.

**Figure 3:**
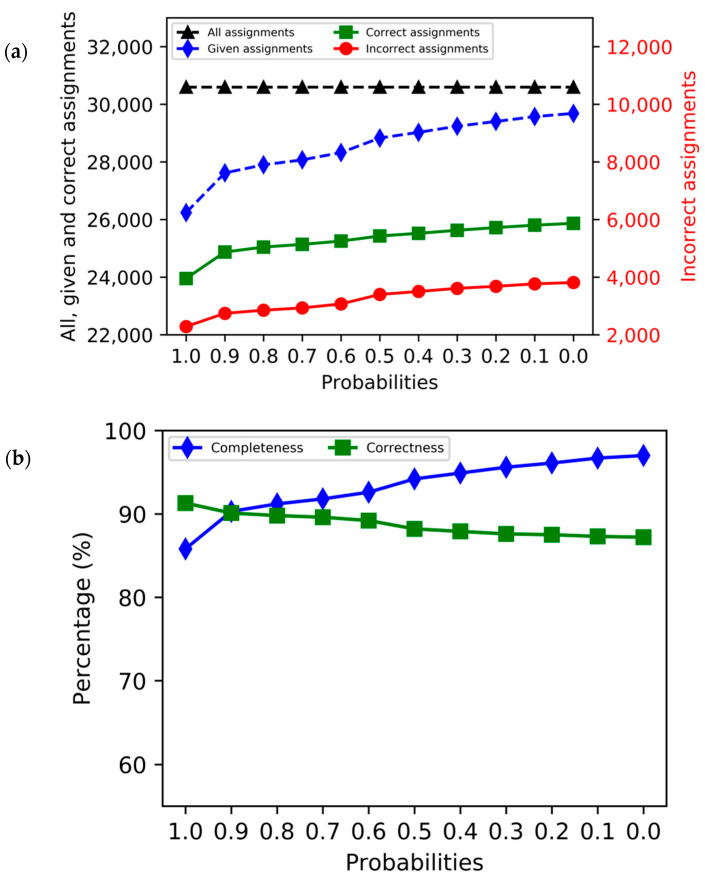
Results from ssPINE analysis of synthetic ssNMR data as averages for the 82 proteins studied. (**a**) Chemical shift assignment probabilities returned by ssPINE for all assignment candidates (x-axis) versus assignment type (y-axis). All (dashed black), given (dashed blue), and correct (solid green) assignments are represented by the numbers on the left side, whereas the incorrect assignments (solid red) are represented by the numbers on the right side. (**b**) Data from the assignment candidate for each protein with the highest assignment probability. Completeness (solid blue) and correctness (solid green) are plotted as a function of that assignment probability.

The total number of assignment candidates returned by ssPINE are plotted as a function of their probability scores in Figure 3a. They are shown as “correct”, “incorrect”, “given” (sum of correct and incorrect), and “all”. The “all” category includes “given” plus invalid assignments, namely those with scores below the probability cutoff.

The correctness and completeness parameters for all assignment candidates with the highest probability for each protein are plotted with respect to their probability in Figure 3b. The correctness decreased moderately as a function of lower probability. The fact that it remained above 85% means that more than 85% of the given chemical shift values were assigned correctly. Overall, the completeness ranged between 85% and 97%. The completeness increased abruptly between 1.0 and 0.9 probability, and then more gradually to 0.0 probability. Plots of percentages of completeness versus correctness for each BMRB entry at each probability are given in SI Figure S2.

The unrefined GB1 peak lists led to a few incorrect ^13^C^α^ assignments (Figure 2a) because false signals picked by automated peak picking algorithm were close to the BMRB average chemical shift value. Manual refinement of the peak lists alleviated this problem by removing false-positive peaks, adding unpicked peaks, and resolving overlaps.

Of the 82 synthetic sets of peak lists analyzed by ssPINE, only three yielded assignment correctness below 70% with a probability cutoff of 0.5. These are denoted by red circles in SI Figure S2 and by red text in SI Table S1. One of the poorest scoring datasets (completeness = 84.5% (474/561); correctness = 66.9% (317/474)) corresponded to BMRB entry 15,716 (the AlgE6R1 subunit from the *Azotobacter vinelandii* Mannuronan C5-epimerase), a 153 amino acid protein containing 27 glycine residues with many overlapping peaks in the carbon alpha region (~45 ppm).

## 4. Discussion

In this report, we have introduced the ssPINE algorithm for the automated analysis and assignment of solid-state

NMR data from membrane proteins and other difficult protein systems. ssPINE builds on the technology of our I-PINE web server for solution NMR data, which serves several thousand jobs annually. We have adapted the I-PINE algorithm to account for the challenging features of ssNMR data from these systems. These include broader lines, extensive inter-residue dipolar interactions, and 2/3D ssNMR experiments that yield a variety of connectivities. As with I-PINE, ssPINE accepts the amino acid sequence of the protein and raw or refined peak lists as an input from a variety of NMR experiments (Table 1). The output of ssPINE includes peak assignments and their probabilities. We have tested and refined the implementation of the ssPINE algorithm with the excellent set of ssNMR data from the small protein, GB1. We also used ssPINE as an input for a set of synthetic peak lists that simulated ssNMR data from 82 other proteins of various sizes that were generated from solution NMR data deposited in BMRB. As shown above, the choice of probability cutoff is an important factor in maximizing correct assignments. In solution NMR, the recommended probability cutoff for I-PINE is 0.5 because it leads to a higher probability of correct assignments [20]. With ssNMR data, a cutoff of 0.6 appears to provide optimal completeness and assignment correctness. Glycine residues are harder to assign because they lack the CB signals that ssPINE uses to evaluate connectivities. Proteins that contain a high glycine content (e.g., BMRB entry 15,716) are particularly problematic because ssPINE has difficulty distinguishing among the several glycine candidates.

Currently, the user can use the ssPINE extension in POKY (two-letter-code *EP*) to generate and submit peak lists from the web browser to the ssPINE webserver. The user can use the *Convert (ss)I-PINE outputs to POKY plugin* in POKY (two-letter-code *ip*) to convert the assigned chemical shift table file from ssPINE to the POKY resonance list file with the chosen probability cutoff. Finally, the POKY Notepad (two-letter-code *Pn*) can be used to propagate assigned peaks onto ssNMR spectra: this is enabled by the script, *Simulate SSNMR peaks with assignments labels* (*predict-and-confirm*).

The analysis of ssNMR data from membrane proteins is highly challenging. ssPINE offers a promising approach for resolving the chemical, structural, and dynamic information contained in these spectra. Information of this kind is crucial for understanding the mechanisms underlying membrane transport, energy transfers, and signaling. We encourage feedback from users of ssPINE, particularly those analyzing ssNMR spectra of membrane proteins, as a means for guiding its further development. Our immediate goals with ssPINE are to incorporate information from strategies commonly used in NMR spectroscopy of membrane proteins, including mutational analysis, ^19^F labeling, and/or selective isotopic labeling.

Longer-term plans are to develop and release a program (ssPINE-POKY) that will include a graphical user interface analogous to that in PINE-SPARKY.2 for solution NMR. In addition, we envision an “integrative” version of ssPINE that will increase assignment correctness and completeness by implementing adaptive probability density functions that incorporate machine learning (ML)-based chemical shift and structure prediction methods, and will provide a comprehensive visualization of structural and dynamic information from ssNMR data, which is analogous to that afforded by I-PINE for solution NMR data.

## 5. Web Server Availability

The usage of the webserver is described in Section 2.5. The web server for ssPINE is freely accessible at https://poky.clas.ucdenver.edu/ssPINE.

## Figures and Tables

**Table 1:** ssNMR experiments supported by ssPINE with their dimensionality and connectivity profiles. CX(i) represents carbon A, B, D, E, G, or H atoms of the ith residue; N(i) represents the nitrogen atom of the ith residue; and CO(i − 1) represents the carbon atom of the carboxyl group of the preceding residue. The minimum set of experiments needed is indicated by asterisks.

Experiment	Dimension	Profile
CC *	2D	CX/O(i)-CX/O(i)
NCA *	2D	N(i)-CA(i)
NCACB	2D	N(i)-CA/B(i)
NCO *	2D	N(i)-CO(i − 1)
NCACO	3D	N(i)-CA(i)-CO(i)
NCACB	3D	N(i)-CA(i)-CA/B(i)
NCACX *	3D	N(i)-CA(i)-CX(i)
NCOCX *	3D	N(i)-CO(i − 1)-CX/C(i − 1)
NCOCA	3D	N(i)-CO(i − 1)-CA(i − 1)
NCOCACB	3D	N(i)-CO(i − 1)-CA/B(i − 1)
CANCO	3D	CA(i)-N(i)-CO(i − 1)
CANCOCX *	3D	CA(i)-N(i)-CX/O(i − 1)
CANCOCA	3D	CA(i)-N(i)-CA/O(i − 1)
CANCOCACB	3D	CA(i)-N(i)-CO/A/B(i − 1)

* Minimum experiments to run ssPINE.

## Data Availability

Not applicable.

## Supplementary Materials

The following are available online at https://www.mdpi.com/article/10.3390/membranes12090834/s1. Figure S1: Flowchart of the ssPINE algorithm; Figure S2: Scatter plots of percentages of completeness verses correctness of each BMRB entry at different probabilities. Red circle indicates the three poorly performing entries: 15716, 15797 and 19755. These are indicated by red text in SI Table S1; Table S1: List of BMRB entries used for the ssPINE performance benchmark. Poorly performing entries (15716, 15797 and 19755) are shown in red.

## References

[1] Ilari A., Savino C. (2008). Protein Structure Determination by X-ray Crystallography. Bioinformatics.

[2] Fujiyoshi Y. (1998). The Structural Study of Membrane Proteins by Electron Crystallography. Adv. Biophys..

[3] Allen O áHill H. (1995). Immobilization of Small Proteins in Carbon Nanotubes: High-Resolution Transmission Electron Microscopy Study and Catalytic Activity. J. Chem. Soc. Chem. Commun..

[4] Wüthrich K. (2001). The Way to NMR Structures of Proteins. Nat. Struct. Biol..

[5] Shuker S.B., Hajduk P.J., Meadows R.P., Fesik S.W. (1996). Discovering High-Affinity Ligands for Proteins: SAR by NMR. Science.

[6] Karsisiotis A.I. (2008). NMR Studies of Inhibitor Binding to Metallo-B-Lactamases. Ph.D. Thesis.

[7] Kempf J.G., Loria J.P. (2002). Protein Dynamics from Solution NMR. Cell Biochem. Biophys..

[8] Kovermann M., Rogne P., Wolf-Watz M. (2016). Protein Dynamics and Function from Solution State NMR Spectroscopy. Q. Rev. Biophys..

[9] Castellani F., Van Rossum B., Diehl A., Schubert M., Rehbein K., Oschkinat H. (2002). Structure of a Protein Determined by Solid-State Magic-Angle-Spinning NMR Spectroscopy. Nature.

[10] Shahid S.A., Bardiaux B., Franks W.T., Krabben L., Habeck M., van Rossum B.-J., Linke D. (2012). Membrane-Protein Structure Determination by Solid-State NMR Spectroscopy of Microcrystals. Nat. Methods.

[11] Hong M. (2000). Solid-State NMR Determination of 13Cα Chemical Shift Anisotropies for the Identification of Protein Secondary Structure. J. Am. Chem. Soc..

[12] Reif B. (2012). Ultra-High Resolution in MAS Solid-State NMR of Perdeuterated Proteins: Implications for Structure and Dynamics. J. Magn. Reson..

[13] De Angelis A.A., Jones D.H., Grant C.V., Park S.H., Mesleh M.F., Opella S.J. (2005). NMR Experiments on Aligned Samples of Membrane Proteins. Methods Enzymol..

[14] Wi S., Frydman L. (2020). An Efficient, Robust New Scheme for Establishing Broadband Homonuclear Correlations in Biomolecular Solid State NMR. ChemPhysChem.

[15] Kobayashi N., Iwahara J., Koshiba S., Tomizawa T., Tochio N., Güntert P., Kigawa T., Yokoyama S. (2007). KUJIRA, a Package of Integrated Modules for Systematic and Interactive Analysis of NMR Data Directed to High-Throughput NMR Structure Studies. J. Biomol. NMR.

[16] Bahrami A., Assadi A.H., Markley J.L., Eghbalnia H.R. (2009). Probabilistic Interaction Network of Evidence Algorithm and Its Application to Complete Labeling of Peak Lists from Protein NMR Spectroscopy. PLoS Comput. Biol..

[17] Lee W., Kim J.H., Westler W.M., Markley J.L. (2011). PONDEROSA, an Automated 3D-NOESY Peak Picking Program, Enables Automated Protein Structure Determination. Bioinformatics.

[18] Lee W., Stark J.L., Markley J.L. (2014). PONDEROSA-C/S: Client–Server Based Software Package for Automated Protein 3D Structure Determination. J. Biomol. NMR.

[19] Lee W., Westler W.M., Bahrami A., Eghbalnia H.R., Markley J.L. (2009). PINE-SPARKY: Graphical Interface for Evaluating Automated Probabilistic Peak Assignments in Protein NMR Spectroscopy. Bioinformatics.

[20] Lee W., Bahrami A., Dashti H.T., Eghbalnia H.R., Tonelli M., Westler W.M., Markley J.L. (2019). I-PINE Web Server: An Integrative Probabilistic NMR Assignment System for Proteins. J. Biomol. NMR.

[21] Lee W., Yu W., Kim S., Chang I., Lee W., Markley J.L. (2012). PACSY, a Relational Database Management System for Protein Structure and Chemical Shift Analysis. J. Biomol. NMR.

[22] Rahimi M., Lee Y., Markley J.L., Lee W. (2021). iPick: Multiprocessing Software for Integrated NMR Signal Detection and Validation. J. Magn. Reson..

[23] Lee W., Markley J.L. (2018). PINE-SPARKY. 2 for Automated NMR-Based Protein Structure Research. Bioinformatics.

[24] Lee W., Rahimi M., Lee Y., Chiu A. (2021). POKY: A Software Suite for Multidimensional NMR and 3D Structure Calculation of Biomolecules. Bioinformatics.

[25] Weber D.K., Wang S., Markley J.L., Veglia G., Lee W. (2020). PISA-SPARKY: An Interactive SPARKY Plugin to Analyze Oriented Solid-State NMR Spectra of Helical Membrane Proteins. Bioinformatics.

[26] Lee W., Tonelli M., Markley J.L. (2015). NMRFAM-SPARKY: Enhanced Software for Biomolecular NMR Spectroscopy. Bioinformatics.

[27] Opella S.J., Marassi F.M. (2004). Structure Determination of Membrane Proteins by NMR Spectroscopy. Chem. Rev..

[28] Gopinath T., Manu V.S., Weber D.K., Veglia G. (2022). PHRONESIS: A One-Shot Approach for Sequential Assignment of Protein Resonances by Ultrafast MAS Solid-State NMR Spectroscopy. ChemPhysChem.

[29] Moseley H.N., Sperling L.J., Rienstra C.M. (2010). Automated Protein Resonance Assignments of Magic Angle Spinning Solid-State NMR Spectra of Β1 Immunoglobulin Binding Domain of Protein G (GB1). J. Biomol. NMR.

[30] Schmidt E., Gath J., Habenstein B., Ravotti F., Székely K., Huber M., Buchner L., Böckmann A., Meier B.H., Güntert P. (2013). Automated Solid-State NMR Resonance Assignment of Protein Microcrystals and Amyloids. J. Biomol. NMR.

[31] Zhou D.H., Nieuwkoop A.J., Berthold D.A., Comellas G., Sperling L.J., Tang M., Shah G.J., Brea E.J., Lemkau L.R., Rienstra C.M. (2012). Solid-State NMR Analysis of Membrane Proteins and Protein Aggregates by Proton Detected Spectroscopy. J. Biomol. NMR.

[32] Gopinath T., Veglia G. (2015). Multiple Acquisition of Magic Angle Spinning Solid-State NMR Experiments Using One Receiver: Application to Microcrystalline and Membrane Protein Preparations. J. Magn. Reson..

[33] Yedidia J.S., Freeman W.T., Weiss Y. (2005). Constructing Free-Energy Approximations and Generalized Belief Propagation Algorithms. IEEE Trans. Inf. Theory.

[34] Bartels C., Xia T., Billeter M., Güntert P., Wüthrich K. (1995). The Program XEASY for Computer-Supported NMR Spectral Analysis of Biological Macromolecules. J. Biomol. NMR.

[35] Keller R.L.J. (2005). Optimizing the Process of Nuclear Magnetic Resonance Spectrum Analysis and Computer Aided Resonance Assignment. Ph.D. Thesis.

[36] Vranken W.F., Boucher W., Stevens T.J., Fogh R.H., Pajon A., Llinas M., Ulrich E.L., Markley J.L., Ionides J., Laue E.D. (2005). The CCPN Data Model for NMR Spectroscopy: Development of a Software Pipeline. Proteins Struct. Funct. Bioinforma..

[37] Eghbalnia H.R., Wang L., Bahrami A., Assadi A., Markley J.L. (2005). Protein Energetic Conformational Analysis from NMR Chemical Shifts (PECAN) and Its Use in Determining Secondary Structural Elements. J. Biomol. NMR.

[38] Wang L., Eghbalnia H.R., Bahrami A., Markley J.L. (2005). Linear Analysis of Carbon-13 Chemical Shift Differences and Its Application to the Detection and Correction of Errors in Referencing and Spin System Identifications. J. Biomol. NMR.

[39] Ulrich E.L., Akutsu H., Doreleijers J.F., Harano Y., Ioannidis Y.E., Lin J., Livny M., Mading S., Maziuk D., Miller Z. (2008). BioMagResBank. Nucleic Acids Res..

[40] Berman H., Henrick K., Nakamura H., Markley J.L. (2007). The Worldwide Protein Data Bank (WwPDB): Ensuring a Single, Uniform Archive of PDB Data. Nucleic Acids Res..

