# Supplementary material for: ssPINE: Probabilistic Algorithm for Automated Chemical Shift Assignment of Solid-State NMR Data from Complex Protein Systems
Source: Membranes (Basel). 2022 Aug 26;12(9):834. doi: 10.3390/membranes12090834 (PMC9503581; doi:10.3390/membranes12090834)
Supplement: Supplementary file 1 [file membranes-12-00834-s001.zip › membranes-1852654-supplementary.pdf]

Supporting Information for

ssPINE: Probabilistic Algorithm for Automated Chemical Shift Assignment of Solid-State NMR Data

Adilakshmi Dwarasala[1], Mehdi Rahimi[1], John L. Markley[1,2], Woonghee Lee[1,*]

[1]Department of Chemistry, University of Colorado Denver, Denver, CO 80217, USA

[2]Department of Biochemistry, University of Wisconsin-Madison, WI 53706, USA

*Correspondence: woonghee.lee@ucdenver.edu

Pseudocode of the ssPINE algorithm

1. Read peak lists from available 2D and 3D ssNMR experiments and the protein sequence as input. The experimental profiles are given in Table 1.

2. Align N, CO, and CX peaks from 2D and 3D spectra based on the reference offsets.

3. Generate spin system matrices with five spins (Figure 1). Different neighboring spins form the system.

    3.1. Form two types of spin systems: one centered on residue (i) and the other on the preceding residue (i-1).

        3.1.1. First, consider the experimental peaks for i^{th} residue spins {N(i) CX(i)}.

        3.1.2. Check all the experimental peaks that refer to (CX(i)) spins (e.g., from NCACX and NCACB peak lists).

        3.1.3. Check to make sure the peak is not a diagonal peak. If it is a diagonal peak, it does not need to be added to the reference spin system list because one axis from NCACX and NCACB probably already has it. If this peak does not exist in the reference spin system list, add it. Fill in any spins missing in the existing spin system. At this point, the reference spin system list has been arranged for the ith residue {N(i) CX(i)} connectivity.

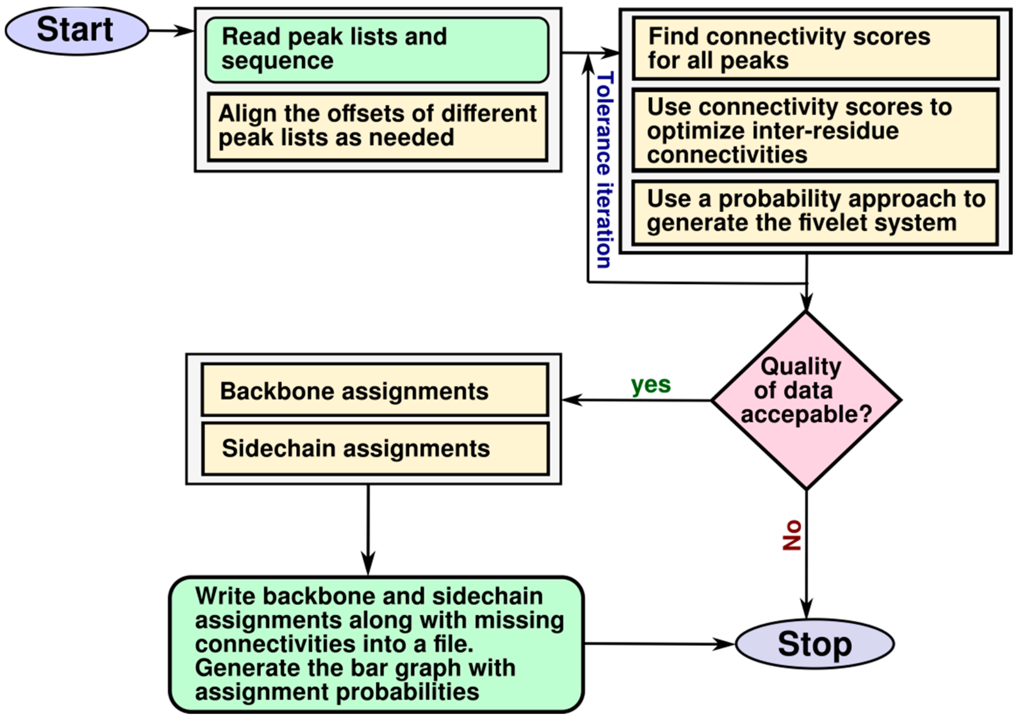

SI Figure S1. Flowchart of the ssPINE algorithm

        3.1.4 Expand the reference spin systems to include inter-spin {N(i) CX(i-1)} connectivities.

    3.2. From the reference spin system, identify triplet spin systems with all possible CX(i) and CX(i-1): {N(i) CA(i) CX(i)} and {N(i) CO(i-1) CX(i-1)}. See Figure 1c.

    3.3. Check the 2D-CC fingerprint to define amenable connectivity score ranges.

3.3.1. From the 2D-CC peak list, check the reliability of CX and fill in missing peaks in the triplet spin systems. Measure the distance correlation coefficient of the carbon spins in both triplet spin systems against 2D-CC peak list (CX(i) from {N(i) CA(i) CX(i)} and CX(i-1) from {N(i) CO(i-1) CX(i-1)}).

$$P = 1 - \left(\frac{r}{\sum r}\right) \quad (1)$$

$$r = \sum_{i=0}^{n} \frac{\min(|x_i - Y|)]}{n} \quad (2)$$

P is distance correlation coefficient, x_i is a chemical shift value of ith element, n is a total number of chemical value elements, Y is a set of chemical shift values of the other strip.

3.4. Calculate the probability of connectivity score with the above coefficients (eq. 1) using the following equation.

$$s(x_q, y_r) = \sum_{m=1}^{q} P_m \prod_{n=1}^{3} f(x_m, y_n) P_n \qquad \forall r \quad (3)$$

$$f(x,y) = \begin{cases} e^{-(\frac{d(x,y)}{C}-1)\times\log(connectivity\ weight)}, & d(x,y) \leq 4C \\ \dfrac{1}{connectivity\ weight}, & d(x,y) > 4C \end{cases} \quad (4)$$

Here, $s(x,y)$ is the connectivity score; Pm and Pn are the distance correlation coefficients of the respective elements; $d(x,y)$ is the Euclidean distance between x and y; and C is the spectral resolution.

3.5. Connect two triplet spin systems {N(i) CA(i) CX(i)} and {N(i) CO(i-1) CX(i-1)} using the connectivity score probabilities (eq. 3) to form a fivelet spin system.

3.5.1. Add connectivity scores to both triplet spin systems and make a few choices for carbon spins that have a score greater than the connectivity weight.

3.5.2. At this point, iterations start to generate more complete spin system matrix. Now refine the spin system matrix using the connectivity scores.

3.5.3. First, take the ith triplet spin system and search for unique choices.

3.5.4. Check for common elements that specify unique choices for the $i - 1^{th}$ triplet system.

3.5.5. Connect those two triplet spin systems that have unique choices for the same element.

3.5.6. If an element does not have a unique choice, then take the ratio of two highest connectivity scores for that element.

3.5.7. If the ratio is greater than the connectivity weight, then connect the two triplet spin systems.

3.5.8. After connecting the two triplet spin systems, remove those element choices from the other triplet system.

3.5.9. The same way, fix the other connectives of triplet spin systems with the connectivity score that has a score greater than the connectivity weight.

3.6. Derive probabilities for assignment of the CA, CB, and C from CX by using the BMRB chemical shift statistics for different residue types.

3.7. Now we have a fivelet spin systems (N CA CB CO N).

3.8. Optimize the fivelet spin system using the probabilistic approach.

3.8.1. Identify an optimal fivelet spin system based on similarity scores ((x,y) given in Eq. 5).

$$(x, y) = \begin{cases} e^{-d(x,y)\times\log(connectivity\ weight)}, & d(x,y) \leq 4C \\ \dfrac{1}{connectivity\ weight}, & d(x,y) > 4C \end{cases} \tag{5}$$

Here, $d(x, y)$ is the Euclidean distance between x and y and C is the spectral resolution.

3.8.2. Cross-check this optimized fivelet spin system against other experimental peak values (e.g., from CAN-COCX) and remove duplicates. Now, an optimal fivelet spin system matrix has been generated with N CA CB CO N.

4. Calculate the data quality by (1) the number of established spin systems and (2) the number of probable connective spin systems. If the data quality meets the goal, then ssPINE moves on to the assignment process. Otherwise, the algorithm stops and ssPINE notifies the user that more data are needed.

Backbone and sidechain assignment routines are similar to those in PINE/I-PINE.

5. Backbone assignment routine.

5.1. Generate N/CA/CO spin system assignments by solving a weighted bipartite graph. Find the marginal probability of assigning the spin system to the sequence (pentapeptide) using the Belief propagation algorithm.

5.2. Calculate secondary structures from the chemical shift assignments.

5.3. Detect outliers and perform reference corrections.

5.4. Iterate steps 5.1-5.3 to find the best marginal probability to assign the amino acids until it reaches the maximum number of iterations or the convergence.

5.5. Perform assignment of individual backbone atoms.

6. Sidechain assignment.

6.1. Prepare initial probably density functions for each sidechain atom type.

6.2. Perform Bayesian updates using available experiments given by the user as inputs.

7. Write a file containing the backbone, sidechain assignments, and missing connectives. Create a spin-system probability bar graph.

8. Compress the results into a zip file and send them to the user.

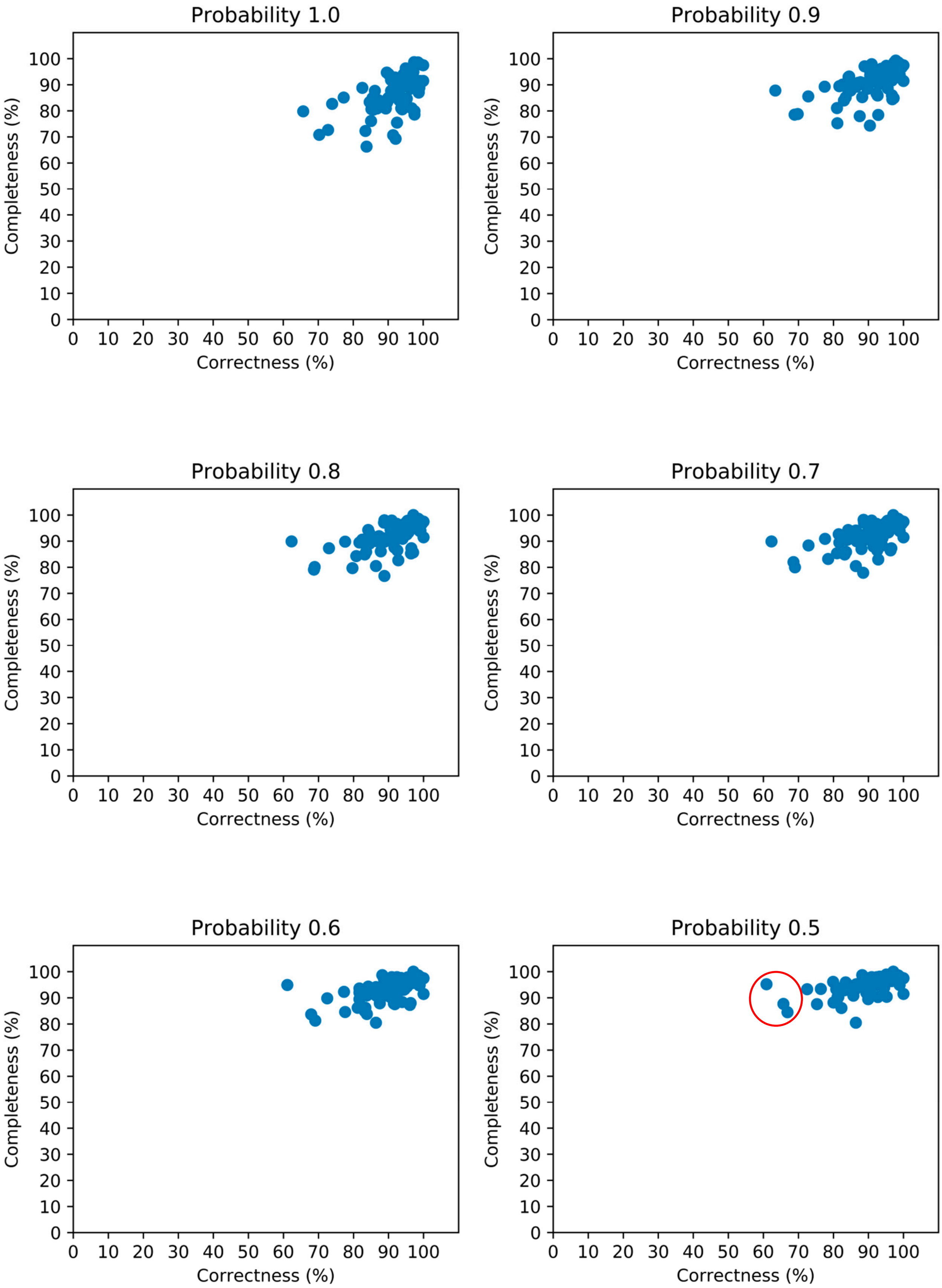
Probability 1.0
Probability 0.9
Probability 0.8
Probability 0.7
Probability 0.6
Probability 0.5
Completeness (%)
Correctness (%)
0
10
20
30
40
50
60
70
80
90
100

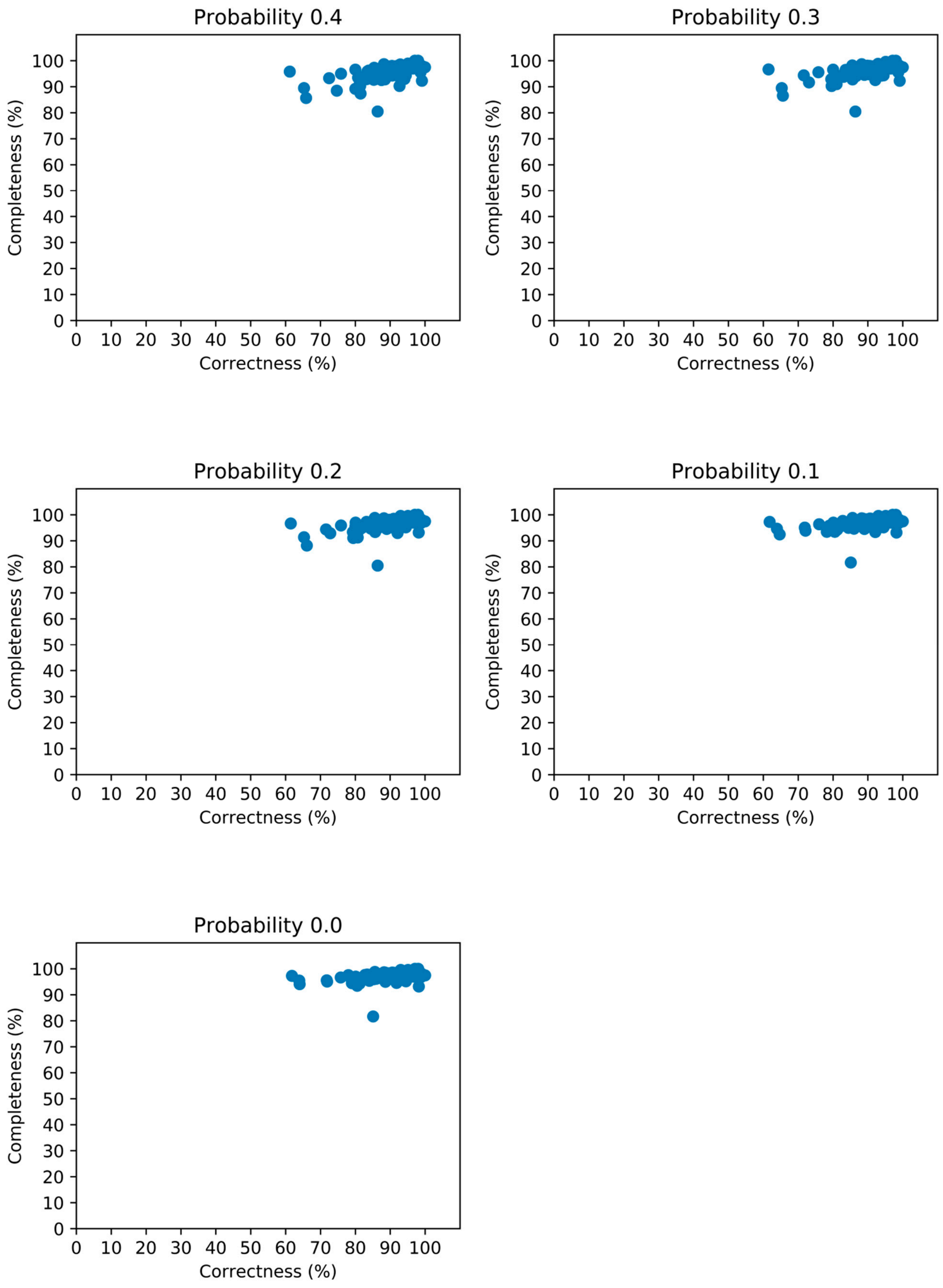

SI Figure S2. Scatter plots of percentages of completeness verses correctness of each BMRB entry at different probabilities. Red circle indicates the three poorly performing entries: 15716, 15797 and 19755. These are indicated by red text in SI Table 1.

SI Table S1. List of BMRB entries used for the ssPINE performance benchmark. Poorly performing entries (15716, 15797 and 19755) are shown in red.

No.	BMRB entry #	Size (a.a.)	Completeness (%) [1]	Correctness (%) [1]	Sequence
1	19041	37	100.0	97.1	EKTNLEIIILVGTAVIAMFFWLLLVIILRTVKRANGG
2	5480	148	97.8	93.5	ADQLTEEQIAEFKEAFSLFDKDGDGTITTKELGTVM RSLGQNPTEAELQDMINEVDADGNGTIDFPEFLTM MARKMKDTDSEEEIREAFRVFDKDGNGYISAAELR HVMTNLGEKLTDEEVDEMIREADIDGDGQVNYEEF VQMMTAK
3	17965	110	97.8	95.9	MTGQELRQLLLDKWGYSYDVQFRRTQGKIFLQVM WKYLEQASFPMNETEYQEHLDSVANYLHALGGAV QVKTFITQTKERPRLGKAVSIPLDLGERASEWIILEH HHHHH
4	16478	104	97.5	90.2	GSAMADIGSEFEKTPSLTRRIDDTIFQNPMVQEAIR MGFSFKDIKKIMEEKIQISGSNYKSLEVLVADLVNAQ KDSMQDESSQTSLQKEISTEEQLRRLQEEKL
5	6498	77	95.4	91.2	MASVLEELQKDLEEVKVLLEKSTRKRLRDTLTSEKSKI ETELKNKMQQKSQKKPELDNEKPAAVVAPLTTGYT VKIS
6	16029	94	94.8	89.7	MGHHHHHHSHAAVRKIHVTVKFPSKQFTVEVDRT ETVSSLKDKIHIVENTPIKRMQLYYSGIELADDYRNLN EYGITEFSEIVVFLKSINRAKD
7	4340	162	88.2	80.0	EEASSTGRNFNVEKINGEWHTIILASDKREKIEDNG NFRLFLEQIHVLEKSLVLKFHTVRDEECSELSMVADK TEKAGEYSVTYDGFNTFTIPKTDYDNFLMAHLINEK DGETFQLMGLYGREPDLSSDIKERFAQLCEEHGILRE NIIDLSNANRCLQARE
8	17611	142	92.5	85.5	MNITVETTVAAPVGKVWRAYTTPEDIKQWNAASD DWHTTAATVDLREGGAFSSRMEAKDGSMGFDFAG TYTKVVENKRIEYAFGDRTAKVEFLEAPQGVTVRVSF VAETEYPVEQQQQGWQAILNNFKRHVESHLEHHH HHH
9	25790	69	98.9	95.1	MQQKQQIMAALNSQTAVQFQQYAAQQYPGNYEQ QQILIRQLQEQHYQQYMQQLYQVQLAQQQAALQK QQ
10	16319	60	93.9	94.5	LVAYGIAQGTAEKVVSLINAGLTVGSIISILGGVTVGLS GVFTAVKAAIAKQGIKKAIQL
11	15407	64	97.9	92.4	GAMGAKEYCRTLFPYTGTNEDELTFREGEIIHLISKET GEAGWWKGELNGKEGVFPDNFAVQIS
12	5539	72	92.8	86.0	MYLTLQEWNARQRRPRSLETVRRWVRESRIFPPPV KDGREYLFHESAVKVDLNRPVTGSLLKRIRNGKKAK S
13	16277	119	90.4	95.2	SEALAKLISLQATEATIVTLDSDNILLSEEQVDVELVQR GDIIKVVPGGKFPVDGRVIEGHSMVDESLITGEAMP

					VAKKPGSTVIAGSINQNGSLLICATHVGADTTLSQIVKLVEEAQ
14	16629	151	97.3	89.8	MQNHDLESIKQAALIEYEVREQGSSIVLDSNISKEPLEFIIGTNQIIAGLEKAVLKAQIGEWEEVVIAPEEAYGVYESSYLQEVPRDQFEGIELEKGMSVFGQTEDNQTIQAIIKDFSATHVMVDYNHPLAGKTLAFRFKVLGFREVSEEE
15	19707	90	97.7	96.1	GSETRTISSTAQERVDLEAVRLASIVDSRLIGTGSVDEDFLREQIRDARYAVIRIPGQPVVEVGTKPTGDVLQGRATGEEGETVLVEEPR
16	11043	70	95.9	92.3	MDETGKELVLALYDYQEKSPREVTMKKGDILTLLNSTNKDWWKVEVKITVNGKTYERQGFVPAAYVKKLD
17	15821	74	97.2	92.8	MDVEPGKFYKGVVTRIEKYGAFINLNEQVRGLLRPRDMISLRLENLNVGDEIIVQAIDVRPEKREIDFKYIPLE
18	16249	140	95.9	83.5	MASMTGGQQMGRGSMGAASAAVSVGGYGPQSSSAPVASAAASRLSSPAASSRVSSAVSSLVSSGPTNQAALSNTISSVVSQVSASNPGLSGCDVLVQALLEVVSALVSILGSSSIGQINYGASAQYTQMVGQSVAQALAG
19	25778	76	93.3	73.5	GTPSADQVRYNYTELPNGEYCYTPRRRCTSADQCCRPYDTTAAFHGCGRIWPKDKREKVDRCYICNNEKTLCTSVM
20	25798	145	93.8	86.2	MEGDRQYGDGYLLQVQELVTVQEGLSVHVPCSFSYPQDGWTDSDPVHGYWFRAGDRPYQDAPVATNNPDREVQAETQGRFQLLGDIWSNDCSLSIRDARKRDKGSYFFRLERGSMKWSYKSQLNYKTKQLSVFVTALTHGSLVPR
21	17957	145	97.9	95.4	MSKIEEFLTAEEEKAIVDAIRDAEKNTSGEIRVHLEKTSEIDVFDRAMDVFHNLKMDNTKLQNGVLIYVAVEDKTFVIYGDKGINDVVSDDFWDTTRNAIQLQFKQGNFKQGLVDGIEKAGMALAKYFPWKKDDIDELPNTISKG
22	19974	145	97.9	95.4	MSKIEEFLTAEEEKAIVDAIRDAEKNTSGEIRVHLEKTSEIDVFDRAMDVFHNLKMDNTKLQNGVLIYVAVEDKTFVIYGDKGINDVVSDDFWDTTRNAIQLQFKQGNFKQGLVDGIEKAGMALAKYFPWKKDDIDELPNTISKG
23	34002	69	96.2	93.7	AYRPSETLCGGELVDTLQFVCGDRGFYFNRPASRVSRRSPQRGIVEECCFRSCDLALLETYCATPAKSE
24	15825	55	97.6	99.0	MASPTVITLNDGREIQAVDTPKYDEESGFYEFKQLDGKQTRINKDQVRTVKDLLE
25	16260	126	89.5	89.9	MEQETLEQVHLTEDGGVVKTILRKGEGGEENAPKKGNEVTVHYVGKLESSGKVFDSSRERNVPFKFHLGQGEVIKGWDICVASMTKNEKCSVRLDSKYGYGEEGCGESIPGNSVLIFEIELISFRE
26	16907	30	80.5	86.4	GLFGAIAGFIEGGWTGMIDGWYGSGKKKKD

27	25799	145	90.8	85.7	MEGDRQYGDGYLLQVQELVTVQEGLSVHVPCSFSYPQDGWTDSDPVHGYWFRAGDRPYQDAPVATNNPDREVQAETQGRFQLLGDIWSNDCSLSIRDARKRDKGSYFFRLERGSMKWSYKSQLNYKTKQLSVFVTALTHGSLVPR
28	11507	159	94.4	90.2	GKNTSGDLSQKQALQLALSAREHFWNTMSGHNPKVKKAVCPSGTFEYQNLQYVYMCSDLGTKAKAVNYLTPIFTKTAIEKGFKDYHFTVSKGKLAVPIGDGDNLLNWKKSTAKLISKKGSTITYEFTVPTLDGSPSAKRKVTFVKENKKWKVNQFDAVI
29	17955	37	97.8	99.3	KYYGNGVHSTKSGSSVNWGEAFSAGVHRLANGGNGFW
30	15512	108	94.9	93.9	GGSNATAPFFEMKLKHYKIFEGMPVTFTCRVAGNPKPKIYWFKDGKQISPKSDHYTIQRDLDGTCSLHTTASTLDDDGNYTIMAANPQGRVSCTGRLMVQAVNQRGRS
31	6335	62	98.7	97.4	MDVEFKCTCSRERCADALKTLPDEEVDSILAEDGEIDMHCDYCGNHYLFNAMDIAEIRNNAS
32	16667	51	98.5	96.9	SEPDEICRARMTHKEFNYKSNVCNGCGDQVAACEAECFRNDVYTACHEAQK
33	19155	85	95.6	83.7	GHMLDSERDKARKEVEEYVKKIVGESYAKSTKKRHTITVALVNELNNIKNEYLNKIVESTSESQLQILMMESRSKVDEAVSKFEK
34	17089	73	95.2	86.5	MGAPPLPPYSAGGREVTMKKGDILTLLNSTNKDWWKVEVNDRQGFVPAAYVKKLDSGTGKELVLALYDYQEKS
35	11026	70	95.2	91.1	MDETGKELVLALYDYQEKSPREVTMKKGDILTLLNSTNKDWWKVEVKATANGKTYERQGFVPAAYVKKLD
36	25774	32	97.5	100.0	GYCAEKGIKCHNIHCCSGLTCKCKGSSCVCRK
37	18852	26	94.9	98.9	GAMATRQARRNRRRRWRERQRAAAAR
38	16468	41	96.7	93.8	SEDCIPKWKGCVNRHGDCCEGLECWKRRRSFEVCVPKTPKT
39	25927	108	95.9	91.2	AVEVEVPGLLTDHTVSSIGHDFYRAFSDKWESDYTGNLTINERPSARAGSWITITVNQDVIFQTFLFPLKRDFEKTVVAALIQTEEALNRRQINQALLSTGDLAHDEF
40	25243	131	96.2	93.9	MKKVMFVSKRNSSRSQMAEGFAKTLGAGKIAVTSSGLESSRVHPTAIAMMEEVGIDISGQTSDPIENFNADDYDVVISLCGCGVNLPPEWVTQEIFEDWQLEDPDGQSLEVFRTVRGQVKERVENLIAKIS
41	16098	147	92.9	83.2	MVISIRRSRHEEGEELVAIWCRSVDATHDFLSAEYRTELEDLVRSFLPEAPLWVAVNERDQPVGFMLLSGQHMDALFIDPDVRGCGVGRVLVEHALSMAPELTTNVNEQNEQAVGFYKKVGFKVTGRSEVDDLGKPYPLLNLAYVGA

42	19205	33	91.5	100.0	GCPQGRGDWAPTSCSQDSDCLAGCVCGPNGFCG
43	30023	130	97.6	92.4	MSALTQILIVEDEPLIAMMLEDFLEVLDKTPVGTVDT VAGALARVEDGGIDAAILDVNLRGGEKSTPVAEALA ARDIPFVFATGGSDDSVDSRFRDRPVLQKPFTMDG VAKALAALLVPRGSVEHHHHHH
44	30025	130	96.5	93.8	MSALTQILIVEDEPLIAMMLEDFLEVLDKTPVGTVDT VAGALARVEDGGIDAAILDVNLRGGEKSTPVAEALA ARDIPFVFATGGSDDSVDSRFRDRPVLQKPFTMDG VAKALAALLVPRGSVEHHHHHH
45	16126	119	98.7	88.2	GSMHEYSVVSSLIALCEEHAKKNQAHKIERVVVGIG ERSAMDKSLFVSAFETFREESLVCKDAILDIVDEKVEL ECKDCSHVFKPNALDYGVCEKCHSKNVIITQGNEM RLLSLEMLAE
46	15371	88	97.7	90.5	MEWLVKKSCCNKQDNRHVLMLCDAGGAIKMIAEV KSDFAVKVGDLLSPLQNALYCINREKLHTVKVLSASS YSPDEWERQCKVAGKTQ
47	25634	52	97.9	90.9	GTIDEWLLKEAKEKAIEELKKAGITSDYYFDLINKAKT VEGVNALKDEILKA
48	15716	153	84.5	66.9	AQGTDGNDVLIGSDVGEQISGGAGDDRLDGGAGD DLLDGGAGRDRLTGGLGADTFRFALREDSHRSPLGT FSDLILDFDPSQDKIDVSALGFIGLGNGYAGTLAVSLS ADGLRTYLKSYDADAQGRSFELALDGNHAATLSAG NIVFAAATPG
49	16956	181	87.6	75.3	ADPGVEGTPVVGSDLDDELHGTLGSEQILGGGGAD QLYGYAGNDLLDGGAGRDKLSGGEGADTFRFALRE DSHRSPLGTFGDRILDFDPSQDRIDVSALGFSGLGN GYAGSLAVSVSDDGTRTYLKSYEADAQGLSFEVALE GDHAAALSADNIVFAATDAAAAGELGVIGASGQPD DPTV
50	19235	154	93.3	80.8	MVNINHRIGIKASPEKIYQALTTDDGLKKWWTNDIS GAGVVGSTIKFRFNGGGPDFKVTKLIPNKTVCWQH AGNMPESWMGTEISFQLETVENQTFVRFTHSNW HETTDFMAHCNTKWAVFLLSLKDALEIGKGTPFPN DIQIDHSLEHHHHHH
51	25706	84	95.3	94.1	GPGSYTGAGEPSQADLDALLSAVRDNRLSIEQAVTL LTPRRGGGSGGGSMDAKEILTRFKDGGLDRAAAQA LLAGRTPAAAPRP
52	18119	66	96.1	79.9	GPLGSPEETVVIALYDYQTNDPQELALRRNEEYCLLD SSEIHWWRVQDRNGHEGYVPSSYLVEKSP
53	19259	43	96.2	92.7	GLEHMADEEKLPPGWEKRMERSSGRVYYFNHITN ASQWERPSG
54	15125	90	91.0	89.8	GSSGSSGESEEEDKCKPMSYEEKRQLSLDINKLPGEK LGRVVHIIQSREPSLKNSNPDEIEIDFETLKPSTLRELE RYVTSCLRKKRKPQ
55	4928	51	98.4	95.1	ARCKVCRKKGEDDKLILCDECNKAFHLFCLRPALYEV PDGEWQCPACQPAT

56	16996	83	98.1	93.2	MGVLRVGLCPGLTEEMIQLLRSHRIKTVVDLVSADLEEVAQKCGLSYKALVALRRVLLAQFSAFPVNGADLYEELKTSTAILS
57	10213	92	97.6	90.8	GSSGSSGFEPRSLITSDKGFVTMTLESLEEIQDVSCAWKELNRKLSSNAVSQITRMCLLKGNMGVCFDVPTTESERLQAEWHDSDWILSVPA
58	17235	88	94.1	86.8	PGSMVSEEIKAQVMESVIGCLKLNDEQKQILSGTTNLAKDFNLDSLDFVDLIMSLEERFSLEISDEDAQKLETVDDICRYIASKSSDA
59	17074	134	91.2	94.3	GSHMKKVMFVCKRNSCRSQMAEGFAKTLGAGKIAVTSCGLESSRVHPTAIAMMEEVGIDISGQTSDPIENFNADDYDVVISLCGCGVNLPPEWVTQEIFEDWQLEDPDGQSLEVFRTVRGQVKERVENLIAKIS
60	17958	37	98.6	98.5	KYYGNGVHLTKSGLSVNWGEAFSAGVHRLANGGNGFW
61	16061	162	94.9	92.5	MTEEIAGFQTSPKAQVQAAFEEIARRSMHDLSFLHPSMPVYYVSDFTLFEGQWTGCVITPWMLSAVIFPGPDQLWPLRKVSEKIGLQLPYGTMTFTVGELDGVSQYLSCSLMSPLSHSMSIEEGQRLTDDCARMILSLPVTNPDVPHAGRRALLFGRRSGENA
62	19945	176	91.5	89.4	MAITGIFFGSDTGNTENIAKMIQKQLGKDVADVHDIAKSSKEDLEAYDILLLGIPTWYYGEAQCDWDDFFPTLEEIDFNGKLVALFGCGDQEDYAEYFCDALGTIRDIIEPRGATIVGHWPTAGYHFEASKGLADDDHFVGLAIDEDRQPELTAERVEKWVKQISEELHLDEILNA
63	25675	89	90.4	81.2	MAGSHHHHHHGSMSEYIRVTEDENDEPIEIPSEDDGTVLLSTVTAQFPGACGLRYRNPVSQCMRGVRLVEGILHAPDAGWGNLVYVVNY
64	11193	70	95.6	98.7	GSSGSSGLEQYVVVSNYKKQENSELSLQAGEVVDVIEKNESGWWFVSTSEEQGWVPATYLEAQNSGPSSG
65	4677	79	97.5	92.8	GVTKEIRIPTLEECDVCHGSGAKPGTQPQTCPTCHGSGQVQMRQGFFAVQQTCPHCQGRGTLIKDPCNKCHGHGRVERS
66	11196	75	94.2	88.9	GSSGSSGKIMIAALDYDPGDGQMGGQGKGRLALRAGDVVMVYGPMDDQGFYYGELGGHRGLVPAHLLDHMSLHGH
67	19427	89	95.1	88.0	MEPPPKLVLDLERLATVPAEKAGPLQRYAATIQSQRGDYNGKVLSIRQDDLRTLAVIYDQSPSVLTEQLISWGVLDADARRAVASHDEL
68	5844	91	96.4	96.6	MKIISISETPNHNTMKITLSESREGMTSDTYTKVDDSQPAFINDILKVEGVKSIFHVMDFISVDKENDANWETVLPKVEAVFELEHHHHHH
69	17890	153	93.7	90.7	MGHHHHHHHHSGDSPAVTLSAGNYIIYNRVLSPRGEKLALTYPGRQRTPVTVSPLDGSSEQAWILRSYDSNSNTWTISPVGSPNSQIGWGAGNVPVVLPPNNYVW

					TLTLTSGGYNIQDGKRTVSWSLNNATAGEEVSIGAD ATFSGRWVIEKV
70	19824	107	95.9	90.0	DDDDDVSFCASVQLHTAVEMHHWCIPFSVDGQPA PSLRWLFNGSVLNETSFIFTEFLEPAANETVRHGCLR LNQPTHVNNGNYTLLAANPCGQASASIMAAFMD NPF
71	15798	167	86.1	82.3	MAEITFKGGPVTLVGQEVKVGDQAPDFTVLTNSLEE KSLADMKGKVTIISVIPSIDTGVCDAQTRRFNEEAAK LGDVNVYTISADLPFAQARWCGANGIDKVETLSDH RDMSFGEAFGVYIKELRLLARSVFVLDENGKVVYAE YVSEATNHPNYEKPIEAAKALVK
72	25083	72	93.7	83.1	MDRIFMTRTEALEFLLKAHQTAVDKIGHPSHKQTPA DHAAIEALDRLLLDVRARRVDQFQINASAAQIIVTD
73	18193	70	90.3	81.4	AGHMKEFRPGDKVVLPPYGVGVVAGIAQRSVSGVS RAYYQVDFPGSRSKAYVPVEAPHSVGLRKALAPEE
74	18044	234	93.6	82.0	MGKIVLIVDDATNGREAVEKYKELKPDIVTMDITMP EMNGIDAIKEIMKIDPNAKIIVCSAMGQQAMVIEAI KAGAKGFIVNTAAVENPSLITQLAQTFGSQAVVVAI DAKRVDGEFMVFTYSGKKNTGILLRDWVVEVEKRG AGEILLTSIDRDGTKSGYDTEMIRFVRPLTTLPIIASGG AGKMEHFLEAFLAGADAAAAASVFHFREIDGRELK EYLKKHGVNLEHHHHHH
75	7381	126	97.7	93.2	MASVERDETREHRIETEIIVDAEDKEERAMGWYYYL DDTLEFPFMGKWKKKSRKTSTIEEKTVEVLGMAPD DECLKDMYVEVADIGGKDDDVYTAKLSDIEAIDVDD DTQEAIADWLYWLARGYKF
76	15797	167	87.7	65.7	MAEITFKGGPVTLVGQEVKVGDQAPDFTVLTNSLEE KSLADMKGKVTIISVIPSIDTGVCDAQTRRFNEEAAK LGDVNVYTISADLPFAQARWCGANGIDKVETLSDH RDMSFGEAFGVYIKELRLLARSVFVLDENGKVVYAE YVSEATNHPNYEKPIEAAKALVK
77	18507	105	89.8	81.2	GAMGIRNSDSVKPTQQARPTVIRWSEGGKEVFISG SFNNWSTKIPLIKSHNDFVAILDLPEGEHQYKFFVDG QWVHDPSEPVVTSQLGTINNLIHVKKSDFEVFD
78	30079	94	93.4	76.4	MTPIEYIDRALALVVDRLARYPGYEVLLSAEKQLQYIR SVLLDRSLDRSALHRLTLGSIAVKEFDETDPELSRALK DAYYVGIRTGRGLKVDLP
79	19755	90	95.2	60.9	MEIERINEHTVKFYMSYGDIEDRGFDREEIWYNRER SEELFWEVMDEVHEEEEFAVEGPLWIQVQALDKGL EIIVTKAQLSKDLDKLVPR
80	15126	147	94.5	87.5	MRLSDYFPESSISVIHSAKDWQEAIDFSMVSLLDKN YISENYIQAIKDSTINNGPYYILAPGVAMPHARPECG ALKTGMSLTLLEQGVYFPGNDEPIKLLIGLSAADADS HIGAIQALSELLCEEEILEQLLTASSEKQLADIISRG
81	17076	134	97.5	88.4	GSHMKKVMFVCKRNSCRSQMAEGFAKTLGAGKIA VTSCGLESSRVHPTAIAMMEEVGIDISGQTSDPIENF

					NADDYDVVISLCGCGVNLPPEWVTQEIFEDWQLEDPDGQSLEVFRTVRGQVKERVENLIAKIS
82	15843	74	90.3	92.7	MAMNGTITTWFKDKGFGFIKDENGDNRYFHVIKVANPDLIKKDAAVTFEPTTNNKGLSAYAVKVVPLEHHHHHH

[1] Completeness and correctness at probability cutoff = 0.5.
